# African Swine Fever Diagnosis in Africa: Challenges and Opportunities

**DOI:** 10.3390/pathogens13040296

**Published:** 2024-04-02

**Authors:** Mary-Louise Penrith, Juanita van Emmenes, Jean N. Hakizimana, Livio Heath, Tonny Kabuuka, Gerald Misinzo, Theophilus Odoom, Abel Wade, Habibata L. Zerbo, Pam D. Luka

**Affiliations:** 1 Department of Veterinary Tropical Diseases, Faculty of Veterinary Science, University of Pretoria, Onderstepoort, Pretoria 0110, South Africa; 2 Transboundary Animal Diseases, Onderstepoort Veterinary Institute, Agricultural Research Council, Pretoria 0110, South Africa; vanheerdenj@arc.agric.za (J.v.E.); heathl@arc.agric.za (L.H.); 3 SACIDS Africa Centre of Excellence for Infectious Diseases, SACIDS Foundation for One Health, Sokoine University of Agriculture, Morogoro P.O. Box 3297, Tanzania; hakizimana.jean@sacids.org (J.N.H.); gerald.misinzo@sacids.org (G.M.); 4 National Livestock Resources Research Institute, National Agricultural Research Organization, Entebbe P.O. Box 295, Uganda; dr.kabuukat@gmail.com; 5 Department of Veterinary Microbiology, Parasitology and Biotechnology, College of Veterinary Medicine and Biomedical Sciences, Sokoine University of Agriculture, Morogoro P.O. Box 3019, Tanzania; 6 Veterinary Services Directorate, Accra Veterinary Laboratory, Accra P.O. Box M161, Ghana; theodoom@yahoo.com; 7 National Veterinary Laboratory (LANAVET), Garoua P.O. Box 503, Cameroon; abelwade@gmail.com; 8 Ministry of Agriculture, Animal and Fisheries Resources, Ouagadougou 03 BP 907, Burkina Faso; habiouermi@gmail.com; 9 Biotechnology Centre, National Veterinary Research Institute, PMB 1, Vom 930103, Nigeria

**Keywords:** African swine fever, veterinary diagnostic laboratories, networks, regional collaboration

## Abstract

The global spread of African swine fever (ASF) in recent decades has led to the need for technological advances in sampling and diagnostic techniques. The impetus for these has been the need to enable sampling by lay persons and to obtain at least a preliminary diagnosis in the field for early control measures to be put in place before final laboratory confirmation. In rural Africa, rapid diagnosis is hampered by challenges that include lack of infrastructure as well as human and financial resources. Lack of animal health personnel, access to affordable means to transport field samples to a laboratory, and lack of laboratories with the capacity to make the diagnosis result in severe under-reporting of ASF, especially in endemic areas. This review summarizes the challenges identified in gap analyses relevant to low- and middle-income countries, with a focus on Africa, and explore the opportunities provided by recent research to improve field diagnosis and quality of diagnostic samples used. Sampling techniques include invasive sampling techniques requiring trained personnel and non-invasive sampling requiring minimal training, sampling of decomposed carcass material, and preservation of samples in situations where cold chain maintenance cannot be guaranteed. Availability and efficacy of point-of-care (POC) tests for ASF has improved considerably in recent years and their application, as well as advantages and limitations, are discussed. The adequacy of existing laboratory diagnostic capacity is evaluated and opportunities for networking amongst reference and other laboratories offering diagnostic services are discussed. Maintaining laboratory diagnostic efficiency in the absence of samples during periods of quiescence is another issue that requires attention, and the role of improved laboratory networking is emphasized. Early diagnosis of ASF is key to managing the disease spread. Therefore, the establishment of the Africa Chapter of the Global African Swine Fever Research Alliance (GARA) increases opportunities for collaboration and networking among the veterinary diagnostic laboratories in the region.

## 1. Introduction

African swine fever (ASF) is a devastating disease of pigs, caused by a unique DNA virus (*Asfarviridae: Asfivirus*). which has been known in domestic pigs in Africa for more than a century. Managing ASF requires early diagnosis so that control measures can be put in place immediately to contain the outbreak and prevent further spread. A model developed in Uganda suggested that responding to an outbreak by increasing biosecurity measures within 14 days of the start of an outbreak would save more than 70 percent of the domestic pigs at risk, while longer delays would result in far greater losses [1]. Early detection enables the successful application of partial rather than massive culling of pigs, which is increasingly recognized as a feasible method to manage outbreaks with minimal loss of livelihoods [2,3,4,5]. In low- and middle-income countries (LMICs) in Africa, where resource-limited smallholder to subsistence-level pig production often predominates, some constraints hamper early diagnosis of ASF. Based partly on the findings of a gap analysis undertaken in Uganda in February 2023, this study explores challenges for rapid ASF detection and response and the opportunities that exist to overcome them. However, the gap analysis identified several policy issues that would need to be addressed to make the best use of the opportunities. These largely impact funding and stem from policies that do not prioritize animal health and livestock production, and the pig sector may be particularly neglected. The policy issues are beyond the scope of this study but it is clear that communication with policy- and decision-makers is key to addressing the challenges facing the diagnosis of ASF in Africa. 

## 2. Challenges for Early Detection and Confirmation of ASF

### 2.1. Detection and Reporting of Outbreaks

African swine fever is among the animal diseases monitored by veterinary services through both passive and active surveillance and is a transboundary disease of concern that is notifiable to the World Organisation for Animal Health (WOAH). However, reports of ASF to WOAH from Africa are low in number considering that ASF is regarded as an important constraint for pig production in most African countries. While most affected African countries are endemic for ASF and therefore only required to report bi-annually unless there is a reason for immediate notification, reports vary from detailed to vague to altogether absent in terms of numbers of outbreaks. Field investigations in Uganda revealed that only a fraction of the outbreaks that were confirmed were reported to WOAH, with ASF simply noted in reports as being “present” [6]. 

Detection of ASF events in most African countries depends largely on passive surveillance that mainly relies on pig value chain actors. Their role only extends to reporting the disease event to an animal health professional. Underreporting of suspected cases of ASF by pig value chain actors is common due to lack of awareness, poor access to animal health services, failure of animal health services to assist in the event of an outbreak, or fear of unfavourable consequences if control measures such as quarantine are implemented [7,8,9,10]. In addition, the propensity for free-range pig rearing systems where pigs are not or only partially confined poses a major challenge for early detection and reporting [6]. Participatory approaches that include local communities and all other stakeholders as well as transdisciplinary research and communication to ensure a proper understanding of their roles and responsibilities in passive surveillance and improving management of ASF are of utmost importance [11]. However, even if suspected ASF is reported, lack of outbreak investigations that result in samples being submitted to laboratories for confirmation of a field diagnosis is a major contributor to underreporting to WOAH. Veterinary services are understaffed and lacking resources in terms of transportation and sample collection and preservation, especially in remote areas with poor infrastructure that are often distant from the nearest diagnostic facility. This means that if samples are taken, they may not reach the laboratory in a fit state to enable a diagnosis. Recent research has proposed and validated innovative sampling alternatives and point-of-care (POC) tests that can address some of these challenges [4], but to date uptake has been minimal in Africa.

### 2.2. Sample Collection, Preservation, and Transportation

Samples of choice for confirmation of ASF are blood from live pigs and spleen and lymph nodes from dead pigs, as the highest loads of virus can be expected in these samples, and they should be kept cold and submitted to the laboratory as soon as possible [12]. Training is required to ensure that the correct samples are obtained, and blood collection from live pigs is sometimes limited by law to qualified animal health officers. Whole blood samples are preferred for detection of ASF virus (ASFV), as lower amounts of virus are present in serum. Acutely sick domestic pigs often succumb to the disease (within 7–10 days) before a humoral response can be detected, limiting the application of antibody-based diagnostic assays in the early detection of the disease [13]. 

Shipment of samples to a diagnostic laboratory is often problematic in African countries where ASF is endemic. Courier services may not be available or may be considered too expensive, and local animal health services if available may lack vehicles and/or fuel to transport of samples. Delivering samples to a laboratory is often opportunistic resulting in significant delays in confirming the presence of ASFV in affected domestic pig populations. For instance, an outbreak in a remote area in Mozambique had been ongoing for six months before there was an opportunity to send samples to the national laboratory at the opposite end of the country enabling ASF to be confirmed [14]. It is essential that unpreserved samples are kept at 4 °C or lower but not frozen to ensure accurate diagnosis. Couriers may be reluctant to carry infectious biological samples even though according to international regulations less rigorous requirements exist to properly packaged samples for diagnostic or research purposes. For ASF, apart from virus cultures, samples are categorized as Category B dangerous goods (IATA Dangerous Goods Regulations) [15]. Laboratories usually lack ready funds to quickly collect samples in case of an ASF outbreak, which makes cases “disappear” before field investigators arrive at the scene.

Finding safer and cheaper ways to transport clinical samples from the field to laboratories through inactivation and elimination of the need for a cold chain to be maintained was identified as a research need. Several methods to reduce infectivity have been described. Under conditions where a cold chain cannot be maintained, the problem can be partially overcome by preserving fresh tissue samples in formol-glycerosaline. Samples preserved this way cannot be used to culture the virus but enable a diagnosis to be made by polymerase chain reaction (PCR) [16]. Viral DNA can be detected by PCR in degraded or formalin-preserved samples, and it persists for long periods after no viable virus is present [17,18,19]. Dried blood samples collected using 3MM filter paper strips or FTA^®^ cards provide a useful alternative to fresh samples and can be used for the detection of both antigens and antibodies [20,21,22], although sensitivity of these samples is slightly lower than that of fresh samples [12]. Dry blood swabs have also been reported to give good results for genome and antibody detection [23]. Dried blood samples are exempt from IATA regulations relating to infectious specimens [15]. Couriers may need to be made aware of these regulations to avoid ASF specimens being refused on the grounds that they are too dangerous to transport, regardless of their nature.

### 2.3. Laboratory Diagnosis of ASF

Laboratory capacity to diagnose ASF has been established in a large number of African countries, usually with assistance from the Food and Agriculture Organization of the United Nations (FAO), the International Atomic Energy Agency (IAEA), the Defense Threat Reduction Agency (DETRA) and other international and non-governmental organizations. The WOAH and FAO designated reference laboratory for ASF in Africa is hosted by the Agricultural Research Council at the Onderstepoort Veterinary Institute (ARC-OVR) in Pretoria, South Africa [24,25]. Suspect ASF cases can be shipped to the regional reference laboratory, which would cover courier and sample testing costs, but unfortunately, several problems exist with distance from much of the remote region. The commonly used tests are PCR to detect viral DNA and ELISA to detect antibodies, while culture of the virus using the haemadsorption technique is performed in WOAH and FAO reference laboratories and research institutions. Mostly, the other laboratories in Africa cannot perform the haemadsorption technique due to the lack of infrastructure including continuous electricity supply [26] for incubators. The direct fluorescent antibody test was used in African laboratories and elsewhere and is still listed by WOAH [13] but has been superseded by PCR and availability of reagents.

In most African countries the capacity to diagnose ASF is usually only available in national veterinary laboratories situated in the capital cities. Capacity may also be available in provincial veterinary laboratories, private facilities, universities, and research institutes, depending on the level of importance of and interest in ASF, as well as availability of funds from public and private sources. These facilities are seldom accessible to smallholder farmers in remote areas and are themselves faced with many challenges.

In LMICs, cost recovery from animal owners for diagnostic testing is a strong disincentive for sample submission. As ASF is a notifiable or controlled disease in most countries according to WOAH requirements for listed diseases, diagnostic tests should be performed free of charge to ensure detection, with costs covered by government, which is often problematic in poorer countries and can limit the number of tests performed. Low sample turnover impacts negatively on the ability of a laboratory to maintain an adequate level of competence, as funds are rarely sufficient to permit a programme of mock testing (proficiency panels) to maintain competence and ensure that tests are working well in the absence of outbreak samples. Sharing samples amongst laboratories within the region with periodic proficiency testing involving the regional reference laboratory would help to maintain diagnostic capacity. As this exercise requires ASF samples to be moved internationally, the necessary reassurances to couriers as mentioned previously would need to be provided.

Improving the stability of diagnostic reagents and panels during shipment and storage, as well as longer shelf life for them, was identified as an issue for diagnostic laboratories. Laboratories with a low sample turnover may need to discard unused reagents that reach their expiry dates, which is uneconomical. Laboratories that are subject to interruptions in the electricity supply without backup will also experience difficulties in maintaining a suitable temperature for reagent and specimen storage, particularly in tropical climates. A reliable backup electricity source that is activated automatically when mains power fails is essential, particularly for reference laboratories. To avoid having an excess of reagents that have to be discarded on expiry, networking amongst laboratories might enable collaborative acquisition and sharing of reagents. Improving the quantity of samples reaching laboratories would go a long way toward resolving the problem and optimizing disease response and reporting. Identifying or developing reagents with a longer shelf life would be helpful in avoiding wastage.

Identifying less resource-intensive ways of conducting laboratory testing and extracting DNA was considered to be a research need. Serological testing is cheaper and requires less specialized facilities than PCR or culture. Serological tests for antigen as well as antibody testing are available, but the antigen ELISA is less sensitive than the PCR or haemadsorption [13,27] and therefore mainly useful in cases of acute ASF. The use of antibodies to diagnose ASF is recommended in infections of longer duration, as antibodies can be detected 7–10 days post-infection and persist for up to three years or longer, and in the absence of a vaccine they are always proof of exposure to the virus [13]. Diagnostic tests designed to detect antibodies against ASFV are only useful for detecting subacute or chronic infections or previous exposure to the virus [28,29,30]. Furthermore, because of their longevity, detection of antibodies does not provide proof of active infection, and they can be detected long after the virus has been cleared. Antibodies to ASFV have frequently been reported in pigs that show no signs of ASF, but the percentage testing positive on PCR has been considerably lower [31,32,33]. Their use in eradication programmes without an additional test to prove the presence of infectious virus is thus not recommended. Some studies in East and Central Africa suggested that indigenous breeds of pigs might not develop antibodies post-infection with ASFV, which would further reduce their value for diagnosis. However, although it was suggested that this might be due to immunogenetic differences in these pigs, the possibility that the sera tested came from pigs sampled prior to antibody production could not be ruled out [34,35]. Methods to simplify DNA extraction as well as diagnostic tests that do not require nucleic acid extraction have been described and may be useful to laboratories in Africa [36,37,38].

Validation of both diagnostic tests and alternative sampling methods in ASF endemic countries was identified as a research need in the region. the European experience in the last century resulted in a strong focus on diagnosing atypical and chronic forms of ASF caused by viruses of low virulence., Even in long-time endemic countries in southern and eastern Africa the majority of ASF outbreaks are characterized by acute manifestations of ASF and can be diagnosed using tests that have been validated elsewhere. The same applies to sampling methods. Validation is particularly important for novel tests or sampling methods that are developed by African laboratories and for tests or sampling methods developed for domestic pigs that are to be used in other species or sample types. For example, reduced specificity for ASF antibody detection by the most widely used ELISA test was reported when used in tissue exudate and haemolysed samples (i.e., suboptimal samples) obtained from wild boars. It was therefore recommended to use a more specific confirmatory test like immunoperoxidase (IPT) for samples that test positive with a conventional ELISA [39]. Validation is generally an expensive process, and the gap analysis indicated that it would be important to remain informed about validation carried out in reference laboratories and to rely on those results. Development of alternative sampling methods for wild suids was considered to be a research need. Based on previous studies [40] a project investigating the use of ropes for sampling oral fluid to detect antibodies in wild suids in South Africa is being conducted by a team from the ASF reference laboratory and the University of Pretoria. However, the complete immunity of warthogs and bushpigs to the pathogenic effects of the ASFV means that they do not shed the virus, so unfortunately conventional lymph node and spleen samples are the only specimens with a possibility of containing ASF viral genome. For early detection of ASFV in domestic pigs, chewing ropes might be a viable alternative that would enable lay persons to collect the samples. Pigs that are infected but not yet showing clinical signs and are active may be shedding ASF in oral fluid therefore applying this method at the first sign of ASF in a herd could assist rapid confirmation [41,42,43]. Information exists on other samples that may be required for trade purposes and surveillance and can easily be obtained, such as meat and meat exudate [44,45,46,47,48]. The concurrence between positive spleen and muscle samples in the Ugandan study [46] indicated that viraemic pigs were presented for slaughter, which is often the case during ASF outbreaks, when farmers rapidly sell their pigs to limit financial losses, as has been reported previously [49,50,51,52,53,54]. This practice poses a risk for the spread of ASF but does offer opportunities for ASF surveillance and earlier diagnosis at points of sale.

Apart from research on ASF, virus isolation (via heamadsorption) as an additional diagnostic tool would be greatly facilitated by replacement of primary cell cultures with one or more stable genetically homogeneous and biologically relevant cell lines to permit titration, isolation, and propagation of ASFV [55]. Development of such cell lines for sharing amongst laboratories was identified as a research need for African laboratories during the gap analysis. It would be practical to investigate the nature and availability of such cell lines that have been developed elsewhere before embarking on attempts to develop them locally. Examples of such cell lines have been described but are scarces [54,55,56,57,58,59].

A gap that was identified was the inability to diagnose coinfections with other pathogens, in particular pathogens causing diseases that are differential diagnoses for ASF, at the same time as testing for ASF. Several assays to simultaneously detect ASF and classical swine fever have been described [60,61,62,63] as well as multiplex assays for simultaneous testing for more than two porcine pathogens [42,64,65,66,67,68,69] but not all are likely to be widely available commercially. Using the methods described in the literature, well-equipped laboratories might be able to develop and validate their tests for simultaneous detection of porcine viruses other than ASF present in their region. This would be useful, as farmers appreciate timely feedback and in the event of a negative ASF test result would still like to have an answer.

There was a strong focus on POCs, which could solve some of the problems identified, although laboratory confirmation of results is still recommended due mainly to lower sensitivity of field tests. Available POCs are discussed in detail elsewhere [4], but they can be successfully applied in outbreaks [70]. There are concerns that the available POCs will not detect infections caused by low virulent ASFV [71,72,73]. The contention that viruses of lower virulence commonly emerge is controversial, as cases where this was confirmed genetically are scarce [74,75]. Co-infection with different ASFV genotypes appears to be rare under natural conditions [76] but a duplex fluorescent quantitative PCR test to detect co-infection with genotype I and II viruses has been reported [77]. There is no doubt that POCs would be a very valuable addition to the tools available for rapid diagnosis of ASF under African conditions and consequent early containment of outbreaks through implementation of simple biosecurity measures.

## 3. Discussion

Global concerns about managing ASF have grown since the introduction of the virus into the Republic of Georgia in 2007 and have increased exponentially since the first report of ASF in China, home to at least half of the world’s pigs population, in 2018. While eradication is still widely considered to be the best option, modern approaches to animal disease control prioritize preservation of livelihoods as well as elimination of infection, seeking alternatives to stamping out large numbers of both sick and healthy animals [78,79]. For this to be feasible, prevention of infection is key and relies on early diagnosis to enable prophylactic measures to prevent spread without massive destruction of pigs [3]. It is also recognized that there are circumstances under which eradication is not achievable, for example where the natural hosts of the virus are present, or where the threat to livelihoods is too great [80,81,82]. In such endemic situations, good management of ASF is essential to enable pig businesses to survive and thrive and is reliant on good diagnostic capacity. Various recent developments in both diagnostic and communication technology provide opportunities to improve both reporting and diagnosis of ASF.

Possibly because ASF has been endemic in most affected African countries for periods ranging from decades to centuries, official reports only reflect a fraction of ASF outbreaks. There is a clear need to improve passive surveillance, disease reporting and investigation of outbreaks. Reluctance to report ASF due to fear of consequences like quarantine and movement bans is not uncommon [7], but in many instances, reporting is simply too difficult. The advent of mobile phones and their wide usage in Africa has greatly facilitated communication [6,83,84,85], but information as to who to inform may not be available in areas that are poorly served in terms of animal health. Clear lines of communication need to be established between pig keepers and other value chain actors and the veterinary services [6,11]. Identifying and integrating other potential sources of information, like extension officers and community leaders, increases the potential for early detection and investigation of ASF outbreaks in remote areas. Monitoring social media was reported to have been useful in gathering information during the outbreak of H7N9 avian influenza in China [86]. Reports of deaths in pigs on social media were a valuable source of information during the 2020 outbreaks of ASF in Papua New Guinea (Dr. A. Britton, personal communication, 2020) [87].

Point-of-care tests (POCs) have considerable potential to enable rapid field diagnosis of ASF [4,37,70]. Although they are less sensitive than conventional PCR, they efficiently detect animals with high viral loads and are therefore extremely infectious and capable of spreading the disease. Detection of an acute outbreak of ASF is extremely important to prevent movement of highly infectious pigs or pork. In areas with limited diagnostic capacity and poor infrastructure, tests that do not require DNA extraction, sophisticated equipment or highly trained personnel are a valuable tool to increase detection of ASF in the field. However, the use of POCs in Africa is limited or absent. The 2023 GARA Gap Analysis recommended field testing of available POCs in African conditions and gaining wider support for their use if they prove efficacious. It is recommended that POCs should be used by animal health personnel and the results, particularly if negative, should be confirmed in a laboratory. However, given the scarcity of veterinary personnel in many African countries, research should be directed towards development of diagnostic kits for smallholder or farm-level use, including immediate testing before buying or selling a pig. Innovative digital interactive training and information dissemination initiatives that have been tested in Uganda [88] could be used to demonstrate the correct use of such kits to the farmers.

Increased collaboration and networking among diagnostic laboratories in the region would improve diagnostic capacity and enable sharing of resources such as surplus equipment or consumables. Sharing information on ASF outbreaks through a harmonized information system would improve surveillance and control and provide early warning of a change in the ASF situation in neighbouring countries and trading partners. Laboratory accreditation often requires that proficiency testing is carried out periodically and could be arranged through the VETLAB laboratory network [89]. Reference laboratories usually provide training, either through short courses or to individuals, which could be publicized via the network. Furthermore, while such training is extremely valuable, visits by experts from the reference laboratory to other laboratories in the region to provide in-house training can be even more valuable to ensure that personnel can apply the skills gained under their normal working conditions. Virtual platforms enable a much higher level of contact amongst laboratories and can be used for problem-sharing and finding joint solutions.

New Generation sequencing can provide valuable information on the evolution of the virus and relatedness of strains and should be available and performed in the advanced laboratories in the region. While it is not practical for routine diagnostics, it becomes extremely important to detect possible illegal introduction of unregistered vaccines, as well as for monitoring any changes in the genome of circulating viruses. Currently, the only attenuated live vaccines are registered and locally available from Navetco Central Veterinary Medicine Company, Vietnam, which offers the first commercial ASF vaccine in the world [90].

Gaining greater support for veterinary laboratories and the diagnostic services they provide is imperative to ensure development and maintenance of capacity. Since ASF is a notifiable or controlled disease, governments need to ensure that adequate diagnostic capacity exists to enable rapid detection and confirmation of these diseases in order to put preventive measures in place and improve reporting to WOAH. Government policy- and decision-makers need to understand that maintaining good veterinary laboratory capacity has high costs but provides services that are essential to support both local and international trade in animals and animal products, with a direct impact on public health and food security. Investing in veterinary laboratory capacity is therefore not optional. There are international and non-governmental bodies that support laboratories, but dependence on donor funding is rarely sustainable in the long term, and this must be made clear to governments that have tended to rely on outside sources to keep laboratories going. At the same time, it should be recognized that cost recovery from clients who use the laboratory services to support their businesses, including commercial farming, is appropriate, and a culture of payment by those who can afford it needs to be developed. If the laboratory provides an efficient and holistic diagnostic service with a short turnaround time and good feedback on results, clients are more likely to be willing to pay for it. On the other hand, rapid diagnosis of outbreaks of notifiable diseases, which often occur in resource-limited contexts, is in the government interest. Subsidizing the cost of these tests by government is important to ensure collaboration from farmers even at subsistence level.

Although notifiable diseases are primarily a government concern, private facilities for diagnosing animal diseases may exist and can be valuable partners to government in the detection of notifiable diseases. Private veterinarians are situated in most countries and can also be valuable partners to the state veterinary services, particularly in urban areas. Client confidentiality is often an obstacle for information sharing by the private sector, but this should not be the case for notifiable diseases. University clinics sometimes provide services at a reduced cost to expand the learning opportunities for their students and may receive animals that are suffering from notifiable diseases. This information is usually shared with the state veterinary services and a good relationship with academic facilities in the region will ensure that this is the case. There are few published records of such cooperation, but a good example was recorded in Thailand. The first indication of ASF in Thailand was a pet miniature pig presented for autopsy at a university in Bangkok, who issued a report that triggered an intensive investigation, and official confirmation of circulation of ASFV was obtained from a swab taken at an abattoir in the neighbouring province [91].

The GARA Africa Chapter (GAC) brings together ASF researchers and expertise from all regions in Africa. It can play an important role in assuring effective and sustainable diagnostic capacity for ASF and other pig diseases. Three regional laboratory networks exist in Africa as listed by FAO, one in West and Central Africa, one in the SADC region, and one in North Africa [24]. Additionally, FAO and WOAH provide information on designated reference laboratories for particular diseases including ASF [24,25]. Considerable attention has been given to building diagnostic capacity in veterinary laboratories in Africa [92]. Through the GAC it should be possible to develop a network that links laboratories throughout the region. A task that has already been identified is to determine existing capacity, particularly at local level, to enable those laboratories with low capacity to be supported by those with medium and high capacity. The GAC is well positioned to ensure benefits from the resources available through GARA as well as FAO, WOAH and IAEA, particularly in terms of training and laboratory evaluation. Being united provides greater opportunities for advocacy to improve official support for veterinary diagnostic laboratories and increase usage of their services to ensure sustainability.
